# Dimethylglycine Sodium Salt Alleviates Intrauterine Growth Restriction-Induced Low Growth Performance, Redox Status Imbalance, and Hepatic Mitochondrial Dysfunction in Suckling Piglets

**DOI:** 10.3389/fvets.2022.905488

**Published:** 2022-06-24

**Authors:** Kaiwen Bai, Luyi Jiang, Tian Wang

**Affiliations:** ^1^College of Animal Sciences and Technology, Nanjing Agricultural University, Nanjing, China; ^2^Institute of Dairy Science, College of Animal Sciences, Zhejiang University, Hangzhou, China

**Keywords:** intrauterine growth restriction, suckling piglets, hepatic, redox status, mitochondrial dysfunction, dimethylglycine sodium salt

## Abstract

This study aimed to investigate the mechanism of redox status imbalance and hepatic mitochondrial dysfunction induced by intrauterine growth restriction (IUGR) and relieve this condition through dimethylglycine sodium salt (DMG-Na) supplementation during the suckling period. Thirty normal birth weight (NBW) and 30 IUGR newborns were selected from 20 sows. Briefly, 1 NBW and 1 IUGR newborn were obtained from each litter of 10 sows, and 10 NBW and 10 IUGR newborns were obtained. Additionally, 2 NBW and 2 IUGR newborns were obtained from each litter of another 10 sows, and 20 NBW newborns were allocated to the N [basic milk diets (BMDs)] and ND (BMDs+0.1% DMG-Na) groups. Furthermore, 20 IUGR newborns were assigned to the I (BMDs) and ID (BMDs+0.1% DMG-Na) groups. The results revealed that the growth performance, serum and hepatic redox status, and hepatic gene and protein expression levels were lower (*P* < 0.05) in the I group compared to the N group. Additionally, supplementation with DMG-Na (ND and ID groups) improved (*P* < 0.05) these parameters compared to the non-supplemented groups (N and I groups). In conclusion, the activity of Nrf2/SIRT1/PGC1α was inhibited in IUGR newborns, and this led to their hepatic dysfunctions. Supplementation with DMG-Na activated Nrf2/SIRT1/PGC1α in IUGR newborns, thereby improving their performance.

## Introduction

Intrauterine growth restriction (IUGR) is defined as small-sized individuals at gestational age possessing weights of below the tenth-centile or the population mean minus 2 standard deviations of a population-based nomogram, and this condition is an important issue in animal husbandry (1). IUGR exerts a permanent stunting effect on the efficiency of nutrient utilization, which results in impaired long-term health. This is probably due to lack of food intake, disease, or oxidative damage (2). Liver dysfunction is associated with growth restriction and metabolic disorders during various periods of life. However, little is known regarding the postnatal effects of IUGR on hepatic function. This should be an important topic, as the liver is one of the most important organs that plays an essential role in the metabolism and transformation of nutrients (3). After birth, slow hepatic development may contribute to slow postnatal growth rates in neonates with IUGR, and altered redox status and hepatic mitochondrial function may negatively affect the growth performance of neonates with IUGR throughout postnatal development and later in adulthood (4).

Mitochondria are crucial in converting nutrients into energy through cellular respiration. Thus, compromised hepatic mitochondrial functions are associated with numerous diseases (5). Nuclear factor erythroid 2-related factor 2 (Nrf2) is crucial in maintaining mitochondrial function and is also involved in the activation of the antioxidant defense system (6). Peroxisome proliferator-activated receptor-γ coactivator-1α (PGC1α) is a coactivator that possesses major pleiotropic functions in mitochondrial biogenesis, as it induces the upregulation of mitochondrial genes both at the nuclear and mitochondrial genome levels (7). Its downregulation is usually accompanied by elevated reactive oxygen species (ROS) that are induced by oxidative damage, and finally, result in the activation of antioxidant defenses system (both enzymatic and non-enzymatic) (7). Sirtuin 1 (SIRT1) is originally described as a factor regulating DNA repair and is highly sensitive to cellular redox status, which is also known to control genomic stability and cellular metabolism (8). Studies have found that SIRT1 physically interacted with and deacetylated PGC1α at multiple lysine sites, thus influencing the redox status and mitochondrial function of cells (9).

Dimethylglycine sodium salt (DMG-Na) can improve the redox status of the body and relieve oxidative damage by scavenging the excessive generated free radicals. This is due to its ability to act as an important material for glutathione synthesis. It was found that DMG-Na can protect the body against oxidative damage by improving the utilization of oxygen, thus improving their performance (10–12). In the present study, loss of SIRT1 activity impaired hepatic mitochondrial function *via* its substrate PGC1α, thus destroying the redox status balance and impairing the performance of IUGR suckling piglets. This work also provides a novel insight that supplementation with dimethylglycine sodium salt relieves hepatic redox status imbalance and mitochondrial dysfunction of IUGR pigs *via* the Nrf2/SIRT1/PGC1α network.

## Materials and Methods

This trial was conducted in accordance with the Chinese guidelines for animal welfare and experimental protocols for animal care and was approved by the Nanjing Agricultural University Institutional Animal Care and Use Committee.

### Experiment Design

In this study, 30 normal birth weight (NBW) newborn piglets (1.53 ± 0.04 kg) and 30 IUGR newborn piglets (0.76 ± 0.06 kg) were selected from 20 sows [Duroc × (Landrace × Yorkshire)] according to a previously described method (13). All sows possessed a similar birth order (3rd) and were fed the same gestating diet that met the National Research Council (NRC, 2012) nutrient requirements. Briefly, one NBW and one IUGR newborn were obtained from each litter of the 10 sows, and 10 NBW newborns (NBW group) and 10 IUGR newborns (IUGR group) were obtained for this study. Additionally, two NBW and two IUGR newborns were obtained from each litter of another 10 sows, and the 20 NBW newborns were allocated to the *N* (basic milk diets) and ND (basic milk diets + 0.1% DMG-Na) groups. Further, 20 IUGR newborns were assigned to the I (basic milk diets) and ID (basic milk diets + 0.1% DMG-Na) groups. The two NBW newborns from one litter were separated into *N* and ND groups, and the two IUGR newborns from one litter were divided into I and ID groups. The newborns were fed milk diets for 7–21 days. DMG-Na (99.9% purity) was obtained from Qilu Sheng Hua Pharmaceutical Co., Ltd., Shandong, China.

Newborns were weighed at 0, 7, 10, 13, 16, 19, and 21 days of age. They were fed warm milk that was collected from their corresponding mother every 2–3 h (~9–10 times daily). Each sow was specially attended by experienced staff to collect milk and calculate their body weight. Before the trial, the corresponding staff visited the sows daily and touched them gently. This was performed to allow the sows to adapt to the presence of staff and to reduce unnecessary stress responses. In this study, we intended to imitate the natural feeding conditions for piglets and fed them individually from bottles for 14 days. The piglets in the four groups (N, ND, I, and ID) were raised in plastic houses (1.5 m × 0.7 m × 0.7 m; environmentally controlled; ambient temperature of 33°C) and were freely provided with water.

### Sample Collection

The blood samples from the selected 10 NBW newborns and 10 IUGR newborns were withdrawn from the precaval vein, and the animals were then stunned by electric shock and subsequently slaughtered by jugular bloodletting within 2 h after birth without suckling. At 21 days of age, 40 suckling piglets from the N, ND, I, and ID groups (10 piglets per group) were weighed prior to euthanization, and the blood samples were withdrawn from the precaval vein. The piglets were then anesthetized *via* electrical stunning and sacrificed by exsanguination, and liver samples were subsequently obtained. The serum samples were separated by centrifugation at 3,500 × g for 15 min at 4°C and then stored at −80°C until analysis. The hepatic tissue was removed from the abdominal cavity immediately after the animal died and was collected for analysis of the indicators described below.

### Serum ALT and AST Study

Serum was individually used to measure the levels of alanine aminotransferase (ALT) and aspartate aminotransferase (AST) using the corresponding assay kit following the manufacturer's instructions (Nanjing Jiancheng Institute of Bioengineering, Jiangsu, China).

### Histological Morphology Study

Hepatic samples fixed in 4% buffered formaldehyde were dried using a graded series of xylene and ethanol and then embedded in paraffin for histological processing. The hepatic samples (5 μm in size) were then deparaffinized using xylene and rehydrated with graded dilutions of ethanol. Slides were stained with hematoxylin and eosin (HE). Ten slides for each sample (middle site of the samples) were prepared, and images were acquired using an optical binocular microscope (14).

Hepatic samples were fixed in 1% (v/v) glutaraldehyde solution and stored in the same solution until processing. After post-fixation for 5 min in 2.5% (w/v) osmium tetroxide, hepatic samples were conventionally processed for visualization *via* transmission electron microscopy and examined in a Philips 420 transmission electron microscope at 80 kV (14).

### Redox Status Study

Hepatic samples were homogenized in 0.9% sodium chloride solution on ice and then centrifuged at 3,500 × g for 15 min at 4°C. Both the serum and supernatant were used to calculate the superoxide dismutase (SOD), glutathione peroxidase (GSH-Px), glutathione (GSH), glutathione reductase (GR), catalase (CAT), and malondialdehyde (MDA) levels using the corresponding assay kit following the manufacturer's instructions (Nanjing Jiancheng Institute of Bioengineering). The protein content was performed using a bicinchoninic acid (BCA) protein assay kit according to the manufacturer's instructions (Nanjing Jiancheng Institute of Bioengineering).

### Mitochondria Redox Status Study

The mitochondria from hepatic samples were obtained according to a previously described method (15). The manganese superoxide dismutase (MnSOD), glutathione peroxidase (GPx), GSH, GR, and γ-glutamylcysteine ligase (γ-GCL) levels in the hepatic mitochondria were measured using an assay kit according to the manufacturer's instructions (Nanjing Jiancheng Institute of Bioengineering).

### Oxidative Damage Study

The ROS level was detected using an ROS assay kit (Nanjing Jiancheng Institute of Bioengineering). Briefly, the hepatic mitochondria were incubated with 10 μM of dichlorodihydrofluorescein diacetate (DCFH-DA) and 10 mmol/L of DNA stain Hoechst 33342 at 37°C for 30 min. The DCFH fluorescence of the hepatic mitochondria was calculated at an emission wavelength of 530 nm and an excitation wavelength of 485 nm using a fluorescence reader (FACS Aria III; BD Biosciences, Franklin Lakes, NJ, USA). The results are expressed as the mean DCFH-DA fluorescence intensity over that of the control. MMP levels were calculated according to the method described by Zhang et al. (16). Briefly, the mitochondria were loaded with 1 × JC-1 dye at 37°C for 20 min and then analyzed by flow cytometry (FACS Aria III). The MMP was measured as the increase in the ratio of green to red fluorescence. The results were shown as the ratio of the fluorescence of aggregates (red) to that of the monomers (green). Protein carbonyls (PC) and 8-hydroxy-2-deoxyguanosine (8-OHdG) within the hepatic samples were measured using their respective assay kits following the manufacturer's instructions (Nanjing Jiancheng Institute of Bioengineering).

The number of apoptotic and necrotic cells was measured using an Alexa Fluor^®^ 488 Annexin V/Dead Cell Apoptosis kit (Thermo Fisher Scientific, Inc., Waltham, MA, USA). Briefly, the hepatic samples were ground with a glass homogenizer, and the cells were washed twice with cool PBS buffer (pH = 7.4) and resuspended (2% suspension) in 1 × annexin-binding buffer. Then, the cell density was determined and cells were diluted in 1 × annexin binding buffer until reaching 1 × 10^6^ cells/ml. A sufficient volume of the cell suspension described above was stained with Annexin V-fluorescein isothiocyanate and propidium iodide (1:9 dilution) staining solution in the dark for 15 min at room temperature. After incubation, the forward scatter of cells was determined, and Annexin V fluorescence intensity was measured in FL-1 with an excitation wavelength of 488 nm and an emission wavelength of 530 nm on a FACS Caliber (BD Biosciences).

### Mitochondrial Electron Transport Chain Complexes Study

The electron transport chain (ETC) complexes I, complexes II, complexes III, complexes IV, and complexes V activity in the hepatic samples were tested using corresponding assay kit according to the manufacturer's instructions (SinoBestBio, Shanghai, China).

### Energy Metabolites Study

The glycogen content (Nanjing Jiancheng Institute of Bioengineering), NAD^+^ and NADH concentration (SinoBestBio), and ATP content (Solarbio, Beijing, China) in the hepatic samples were tested using commercial kits according to the manufacturer's instructions, separately. The mtDNA copy number of the hepatic samples was measured using a real-time fluorescence quantitative polymerase chain reaction (PCR) kit (Tli RNaseH Plus; Takara Bio, Inc., Otsu, Japan). In brief, the 20 μl PCR mixture was composed of 10 μl of SYBR Premix Ex Taq (2 ×), 0.4 μl of upstream primer, 0.4 μl of downstream primer, 0.4 μl of ROX dye (50 ×), 6.8 μl of ultra-pure water, and 2 μl of cDNA template. The sequence of the Mt D-loop gene upstream primer was 5′-AGGACTACGGCTTGAAAAGC-3′, and that of the downstream primer was 5′-CATCTTGGCATCTTCAGTGCC-3′. The length of the target fragment was 198 bp. The sequence of the β-actin upstream primer was 5′-TTCTTGGGTATGGAGTCCTG-3′, and that of the downstream primer was 5′-TAGAAGCATTTGCGGTGG-3′. The length of the target fragment was 150 bp. The amplification of each hepatic sample was performed in triplicate. The fold-expression of each gene was calculated according to the 2^−ΔΔ^Ct method (17), and β-actin was used as an internal standard.

### Quantitative Real-Time PCR Study

Quantitative Real-Time PCR (qPCR) was performed as described previously (17). Total RNA was obtained from the hepatic samples using Trizol Reagent (TaKaRa, Dalian, China) and then reverse-transcribed using a commercial kit (Perfect Real Time, SYBR^@^ PrimeScript^TM^, TaKaRa) following the manufacturer's instructions. The mRNA expression levels of specific genes were quantified *via* real-time PCR using SYBR^@^ *Premix Ex Taq* ^TM^ II (Tli RNaseH Plus) and an ABI 7300 Fast Real-Time PCR detection system (Applied Biosystems, Foster City, CA). The SYBR Green PCR reaction mixture consisted of 10 μl SYBR^@^ *Premix Ex Taq* (2X), 0.4 μl of the forward and reverse primers, 0.4 μl of ROX reference dye (50X), 6.8 μl of ddH_2_O, and 2 μl of cDNA template. Each sample was amplified in triplicate. The fold-expression of each gene was calculated according to the 2^−ΔΔ^Ct method (17), and the β*-actin* gene was used as an internal standard. The primer sequences that were used are listed in Supplementary Table S1.

### Western Blot Study

Total protein was isolated from three hepatic samples per group using a radioimmunoprecipitation assay lysis buffer containing a protease inhibitor cocktail (Beyotime Institute of Biotechnology, Jiangsu, China). The nuclear protein in the HEPATIC samples was extracted using a nuclear protein extraction kit (Beyotime Institute of Biotechnology, Jiangsu, China). The concentrations of total cellular and nuclear proteins in the HEPATIC samples were measured using a bicinchoninic acid protein assay kit (Beyotime Institute of Biotechnology). Antibodies against related proteins were purchased from Cell Signaling Technology (Danvers, MA, USA). Thereafter, equal quantities of protein were resolved by SDS-PAGE and then transferred onto polyvinylidene difluoride membranes. Next, the membranes were incubated with blocking buffer (5% bovine serum albumin in Tris-buffered saline containing 1% Tween 20) for 1 h at room temperature and then probed with primary antibodies (1:1000) against Nrf2 (# 12721S), HO1 (# 82206S), SOD (# 37385S), GSH-Px (# 3286S), Sirt1 (# 9475S), PGC1α (# 2178S), OCLN (# 91131S), ZO1 (# 13663S), Cyt C (# 11940S), mtTFA (# 8076S), Mfn2 (# 9482S), Drp1 (# 8570S), Fis1 (# 84580S), and α-tubulin (# 2125S) overnight at 4°C. Then, the membranes were washed with Tris-buffered saline with 0.05% Tween-20 and incubated with a suitable secondary antibody for 1 h at room temperature. Finally, the blots were detected using enhanced chemiluminescence reagents (ECL-Kit, Beyotime, Jiangsu, China) and subsequent autoradiography. Photographs of the membranes were acquired using the Luminescent Image Analyzer LAS-4000 system (Fujifilm Co.) and then quantified with ImageJ 1.42 q software (NIH, Bethesda, MD, USA).

### Statistical Analysis

The body weight data was conducted using a mixed model with group (G), time (T), DMG-Na (D), and G × T × D as fixed effects and piglets as random effects. If the *P* value of the interaction for G × T × D was <0.05, differences in body weight among the different groups were determined using one-way ANOVA. The values with different superscripts that included a, b, and c (N, ND, I, ID group) were significantly different (*P* < 0.05).

The data for hepatic ALT, AST, redox status, oxidative damage, mitochondrial ETC, energy metabolites, and gene and protein expression among trial A (NBW and IUGR), and trial B (N, ND, I, ID) were analyzed separately. For Trial A, the corresponding data were analyzed using a paired *t*-test. For trial B, the corresponding data were analyzed using a mixed model with group (GB), DMG-Na (D), and GB × D as fixed effects and piglets as random effects. If the *P* value of the interaction GB × D was <0.05, a one-way ANOVA was conducted among the different groups. The values exhibiting different *P-values* (NBW, IUGR group in black color) and a, b, c, d (N, ND, I, ID group in red color) were significantly different (*P* < 0.05). Data are expressed as means with standard deviation. All data were analyzed using Statistical Analysis System software (version 9.1; SAS Institute, Inc., Cary, NC, USA). The statistical difference was significant, and the *P* value was no more than 0.05.

## Results

### Growth Performance

The interaction effects (groups × time × DMG-Na) were significant (*P* < 0.001) for the IBW and FBW traits (Table 1). Body weight values were lower (*P* < 0.05) in the I group than they were in the *N* group. Additionally, the FBW values were increased (*P* < 0.05) in the ND and ID groups relative to those of the *N* and I groups, respectively, during the suckling period. The ADG values were decreased (*P* < 0.05) in the I group relative to those in the N group from 10 to 19 d during the suckling period. Additionally, the ADG values were increased (*P* < 0.05) in the ND and ID groups relative to those in the N and I groups, respectively, during the suckling period.

**Table 1:** Supplementation with DMG-Na improved the growth performance of IUGR suckling piglets^1^.

**Items**	**Treatment^2^**				***P*** **value**						
	***N***	**ND**	***I***	***ID***	***P***_**G**_	***P***_**T**_	***P***_**D**_	***P***_**G × T**_	***P***_**G × D**_	***P***_**T × D**_	***P***_**G × T × D**_
IBW (7 d)	3.48 ± 0.26^a^	–	2.23 ± 0.21^b^	–	<0.05	–	–	–	–	–	–
FBW	7.71 ± 0.58^b^	8.90 ± 0.52^a^	5.92 ± 0.60^c^	7.03 ± 0.57^b^	0.038	–	0.764	–	0.378	–	–
**Average daily body weight gain (kg)**										
7–10 d	0.17 ± 0.03^c^	0.52 ± 0.02^a^	0.22 ± 0.04^c^	0.34 ± 0.05^b^	0.001	<0.001	<0.001	<0.001	<0.001	<0.001	<0.001
10–13 d	0.34 ± 0.01^b^	0.49 ± 0.02^a^	0.20 ± 0.02^d^	0.26 ± 0.01^c^							
13–16 d	0.43 ± 0.03^a^	0.31 ± 0.01^b^	0.29 ± 0.03^b^	0.35 ± 0.03^b^							
16–19 d	0.23 ± 0.01^b^	0.22 ± 0.02^b^	0.22 ± 0.02^b^	0.36 ± 0.01^a^							
19–21 d	0.35 ± 0.05	0.39 ± 0.05	0.44 ± 0.05	0.46 ± 0.07							

1 *Values are expressed as mean ± SD, n = 10. Different superscripts a, b, c (N, ND, I, ID group) represent significant differences (P < 0.05)*.

2 *NBW, normal birth weight newborns; IUGR, intrauterine growth restriction newborns; IBW, initial body weight; FBW, final body weight; N, NBW newborns fed a basic milk diet; ND, NBW newborns fed a basic milk diet plus 0.1% DMG-Na; I, IUGR newborns fed a basic milk diet; ID, IUGR newborns fed a basic milk diet plus 0.1% DMG-Na*.

### Serum ALT and AST Study

**Figure 1:**
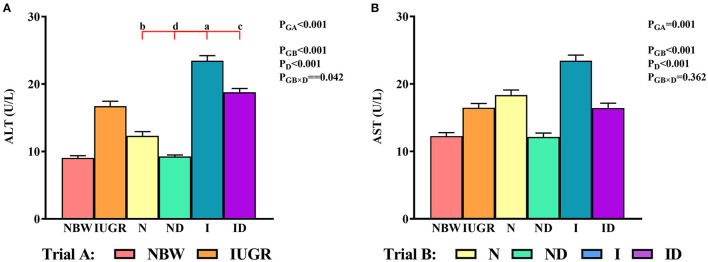
**(A,B)** Supplementation with DMG-Na improved serum ALT and AST concentrations in IUGR suckling piglets. Data are expressed as the mean ± SD; *n* = 10 suckling piglets per group. The *P*-value (Trial A: NBW, IUGR group in black color) and a, b, c, d (Trial B: N, ND, I, ID group in red color) represent significant differences (*P* < 0.05). NBW, normal birth weight newborns; IUGR, intrauterine growth restriction newborns; *N*, NBW newborns fed a basic milk diet; ND, NBW newborns fed a basic milk diet plus 0.1% DMG-Na; I, IUGR newborns fed a basic milk diet; ID, IUGR newborns fed a basic milk diet plus 0.1% DMG-Na. ALT, alanine aminotransferase; AST, aspartate aminotransferase.

In trial A (Figure 1), the serum ALT and AST values in the IUGR group were increased (*P* < 0.05) relative to those in the NBW group. In trial B (Figure 1), the interaction effects (groups × DMG-Na) were significant for ALT (*P* = 0.042). Serum ALT values were decreased (*P* < 0.05) in the ND and ID groups relative to those in the *N* and I groups, respectively. Additionally, serum ALT values were increased (*P* < 0.05) in the I group compared to those in the *N* group.

### Histological Morphology Study

**Figure 2:**
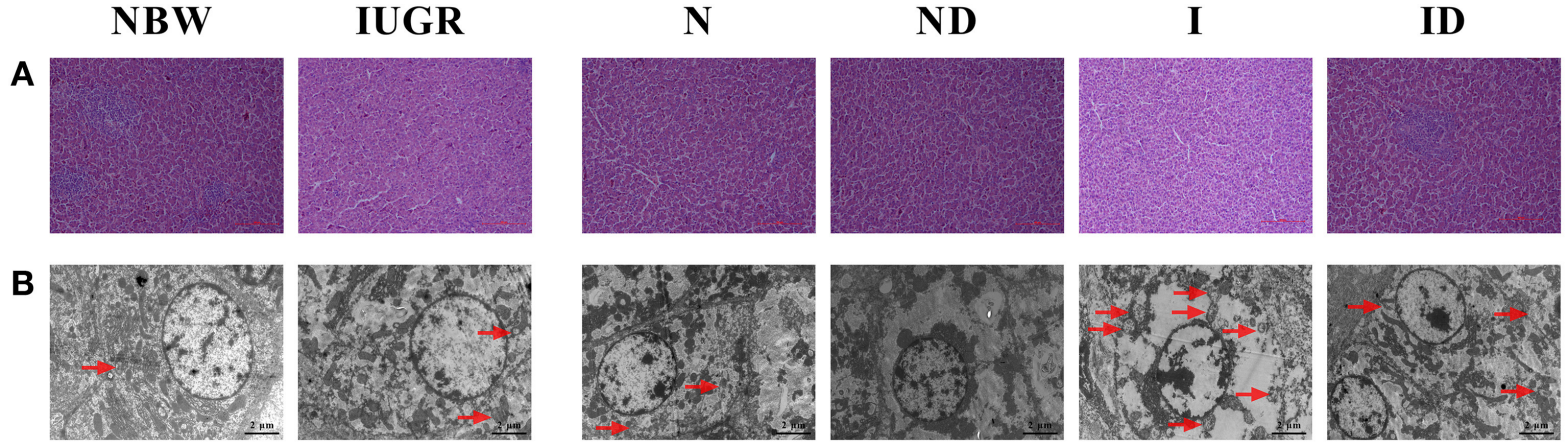
Supplementation with DMG-Na improved the hepatic histological morphology of IUGR suckling piglets. **(A)** Hematoxylin-eosin staining of the liver; Scale bars represent 100 μm. **(B)** Mitochondrial swelling in the liver. Scale bars represent 2 μm. The mitochondria swelling was indicated by the red arrow. NBW, normal birth weight newborns; IUGR, intrauterine growth restriction newborns; N, NBW newborns fed a basic milk diet; ND, NBW newborns fed a basic milk diet plus 0.1% DMG-Na; I, IUGR newborns fed a basic milk diet; ID, IUGR newborns fed a basic milk diet plus 0.1% DMG-Na.

Compared to the NBW group, the livers of the IUGR group were more susceptible to internal structure damage, larger intercellular spaces, and an increased number of mitochondrial swelling (Figure 2). Over time, these phenomena were worse in the N and I group compared with the NBW group and IUGR group. Additionally, the hepatic internal structure damage, larger intercellular spaces, and an increased number of mitochondrial swelling existed in the I group as compared to the N group. Supplementation with DMG-Na group (ND and ID) improved hepatic internal structure, intercellular space, and the number of mitochondrial swelling characteristics compared to those in the N and I group.

### Redox Status Study

**Figure 3:**
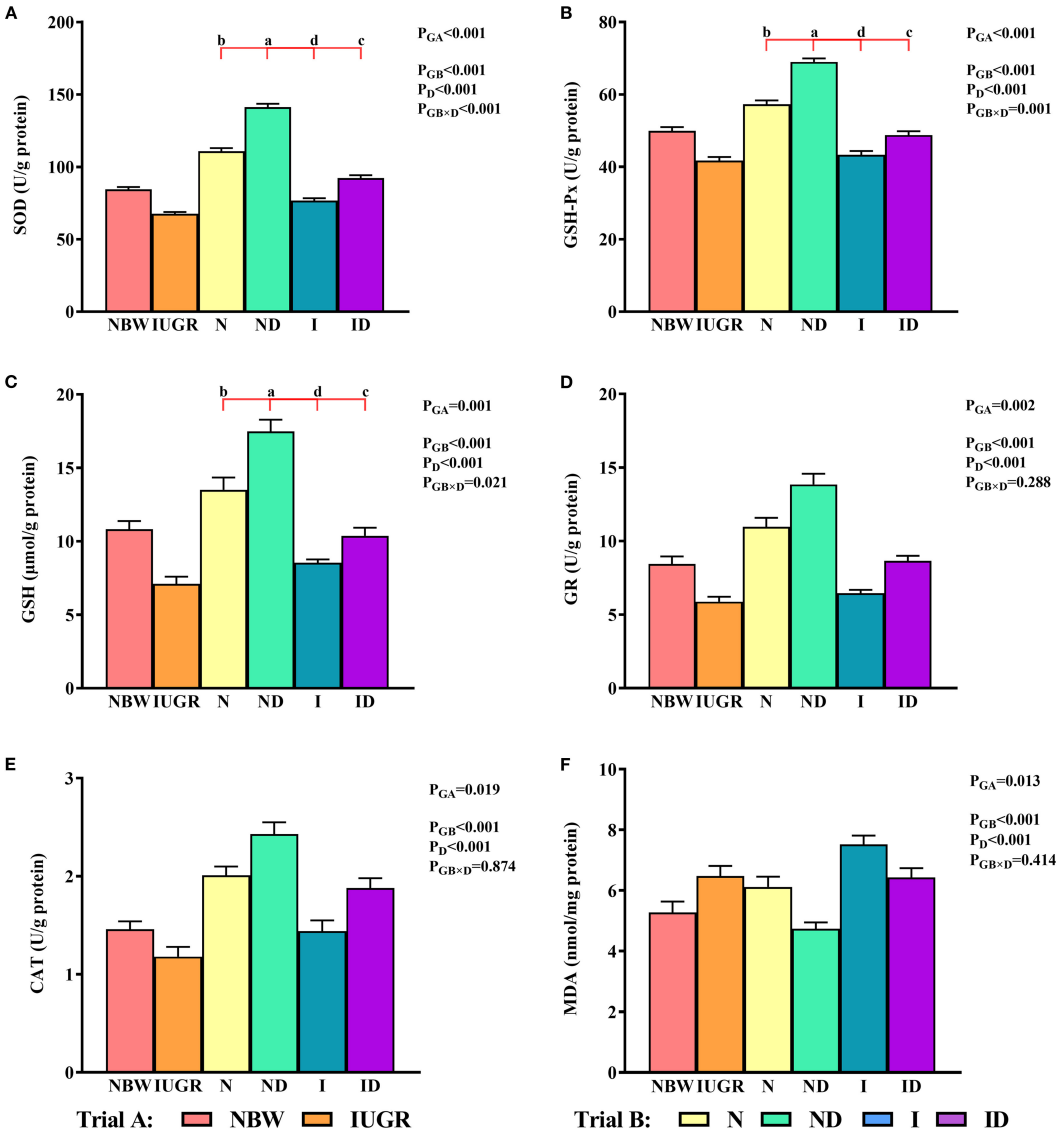
**(A–F)** Supplementation with DMG-Na improved the hepatic redox status of IUGR suckling piglets. Data are expressed as the mean ± SD; *n* = 10 suckling piglets per group. The *P*-value (trial A: NBW, IUGR group in black color) and a, b, c, d (trial B: N, ND, I, ID group in red color) represent significant differences (*P* < 0.05). NBW, normal birth weight newborns; IUGR, intrauterine growth restriction newborns; N, NBW newborns fed a basic milk diet; ND, NBW newborns fed a basic milk diet plus 0.1% DMG-Na; I, IUGR newborns fed a basic milk diet; ID, IUGR newborns fed a basic milk diet plus 0.1% DMG-Na. SOD, superoxide dismutase; GSH-Px, glutathione peroxidase; GSH, glutathione; GR, glutathione reductase; CAT, catalase; MDA, methane dicarboxylic aldehyde.

In trial A (Figure 3), the SOD, GSH-Px, GSH, GR, CAT, and MDA values in the IUGR group were lower (*P* < 0.05) than those in the NBW group. In trial B (Figure 3), the interaction effects (groups × DMG-Na) were significant for SOD (*P* < 0.001), GSH-Px (*P* = 0.001), and GSH (*P* = 0.021). Hepatic SOD, GSH-Px, and GSH levels were increased (*P* < 0.05) in the ND and ID groups relative to those in the *N* and I groups, respectively. Additionally, hepatic SOD, GSH-Px, GR, and CAT values were decreased (*P* < 0.05) in the I group compared to those in the *N* group.

### Mitochondrial Redox Status Study

**Figure 4:**
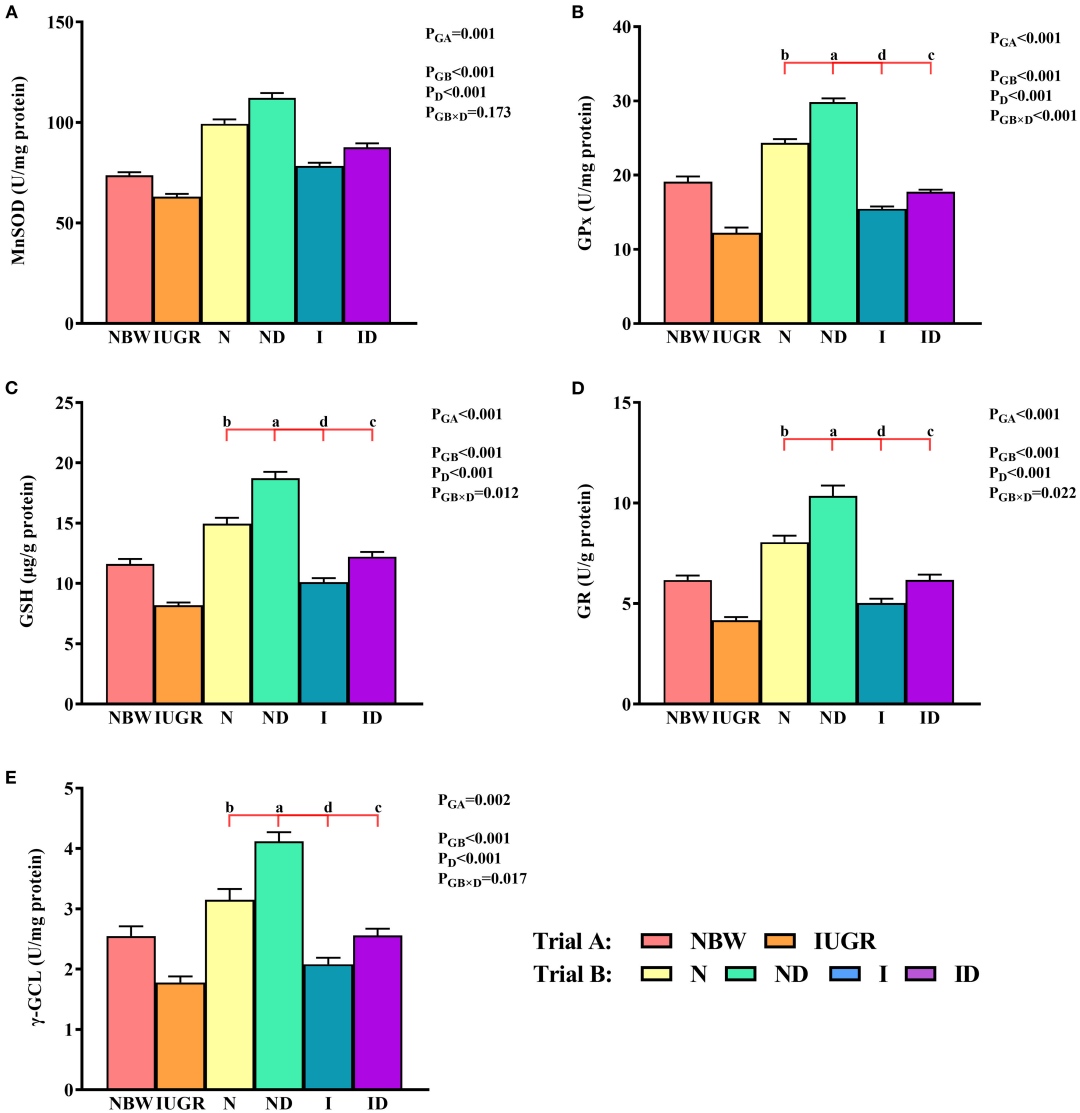
**(A–E)** Supplementation with DMG-Na improved the hepatic mitochondrial redox status of IUGR suckling piglets. Data are expressed as the mean ± SD; *n* = 10 suckling piglets per group. The *P*-value (Trial A: NBW, IUGR group in black color) and a, b, c, d (Trial B: N, ND, I, ID group in red color) represent significant differences (*P* < 0.05). NBW, normal birth weight newborns; IUGR, intrauterine growth restriction newborns; N, NBW newborns fed a basic milk diet; ND, NBW newborns fed a basic milk diet plus 0.1% DMG-Na; I, IUGR newborns fed a basic milk diet; ID, IUGR newborns fed a basic milk diet plus 0.1% DMG-Na. MnSOD, manganese superoxide dismutase; GPx, glutathione peroxidase; GSH, glutathione; GR, glutathione reductase; γ-GCL, γ-glutamylcysteine ligase.

In trial A (Figure 4), the mitochondrial MnSOD, GPx, GSH, GR, and γ-GCL values in the IUGR group were lower (*P* < 0.05) than those in the NBW group. In trial B (Figure 4), the interaction effects (groups × DMG-Na) were significant for GPx (*P* = 0.018), GSH (*P* = 0.012), GR (*P* = 0.022), and γ-GCL (*P* = 0.017). Hepatic mitochondrial GPx, GSH, GR, and γ-GCL values were increased (*P* < 0.05) in the ND and ID groups relative to those in the N and I groups, respectively. Additionally, hepatic mitochondrial MnSOD, GPx, GSH, and GR values were decreased (*P* < 0.05) in the I group relative to those in the N group.

### Oxidative Damage Study

**Figure 5:**
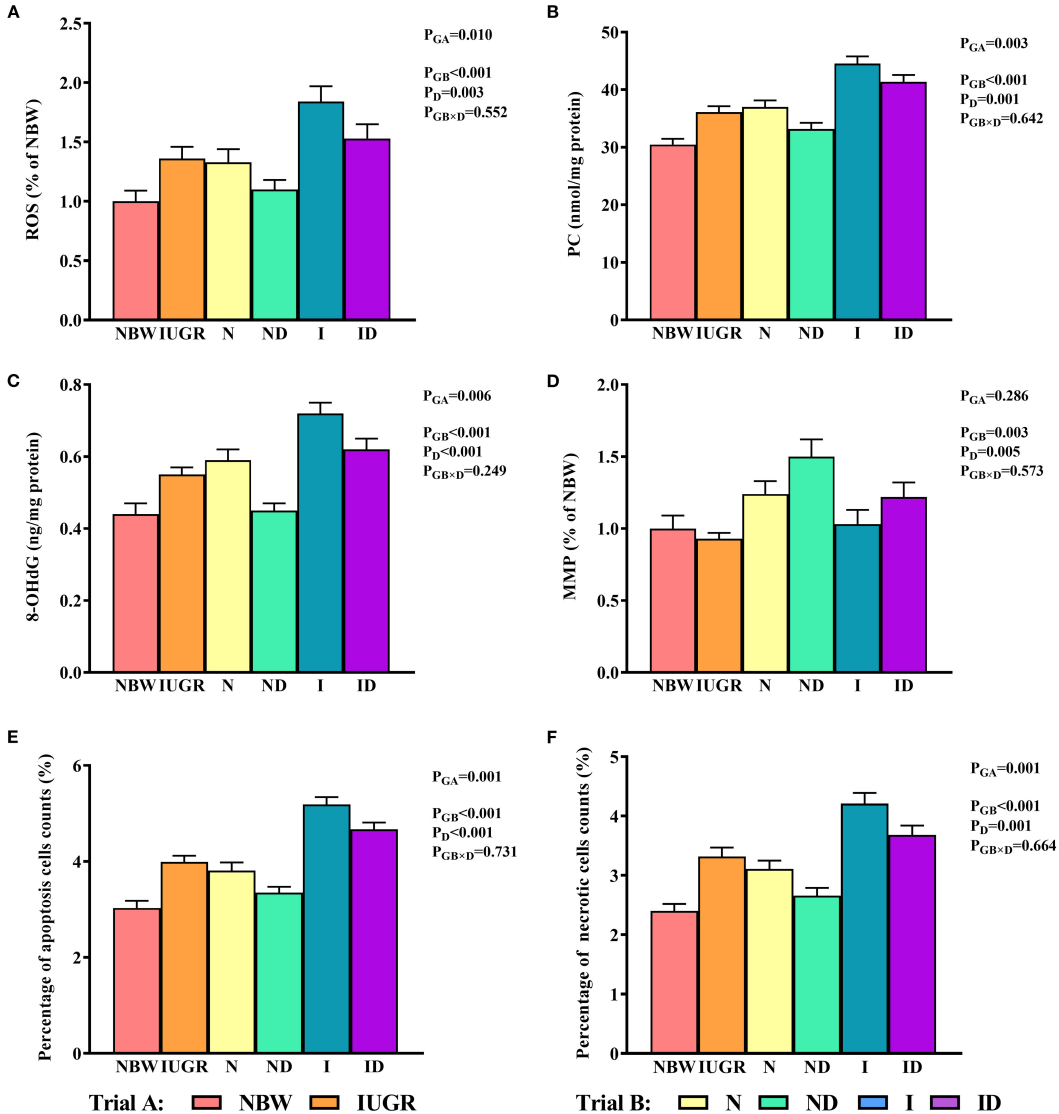
**(A–F)** Supplementation with DMG-Na improved the hepatic oxidative damage of IUGR suckling piglets. Data are expressed as the mean ± SD; *n* = 10 suckling piglets per group. The *P*-value (Trial A: NBW, IUGR group in black color) and a, b, c, d (Trial B: N, ND, I, ID group in red color) represent significant differences (*P* < 0.05). NBW, normal birth weight newborns; IUGR, intrauterine growth restriction newborns; N, NBW newborns fed a basic milk diet; ND, NBW newborns fed a basic milk diet plus 0.1% DMG-Na; I, IUGR newborns fed a basic milk diet; ID, IUGR newborns fed a basic milk diet plus 0.1% DMG-Na. ROS, reactive oxygen species; PC, protein carbonyls; 8-OHdG, 8-hydroxy-2-deoxyguanosine; MMP, mitochondrial membrane potential.

In trial A (Figure 5), the hepatic ROS, PC, 8-OHdG, and percentage of apoptosis and necrotic cell counts in the IUGR group were decreased (*P* < 0.05) relative to those in the NBW group. In trial B (Figure 5), the interaction effects (groups × DMG-Na) were not significant (*P* > 0.05) for any of the tested oxidative damage traits. Additionally, the percentage of apoptotic cells in the liver was increased (*P* < 0.05) in the I group relative to that in the *N* group.

### Mitochondrial ETC Complexes Study

**Figure 6:**
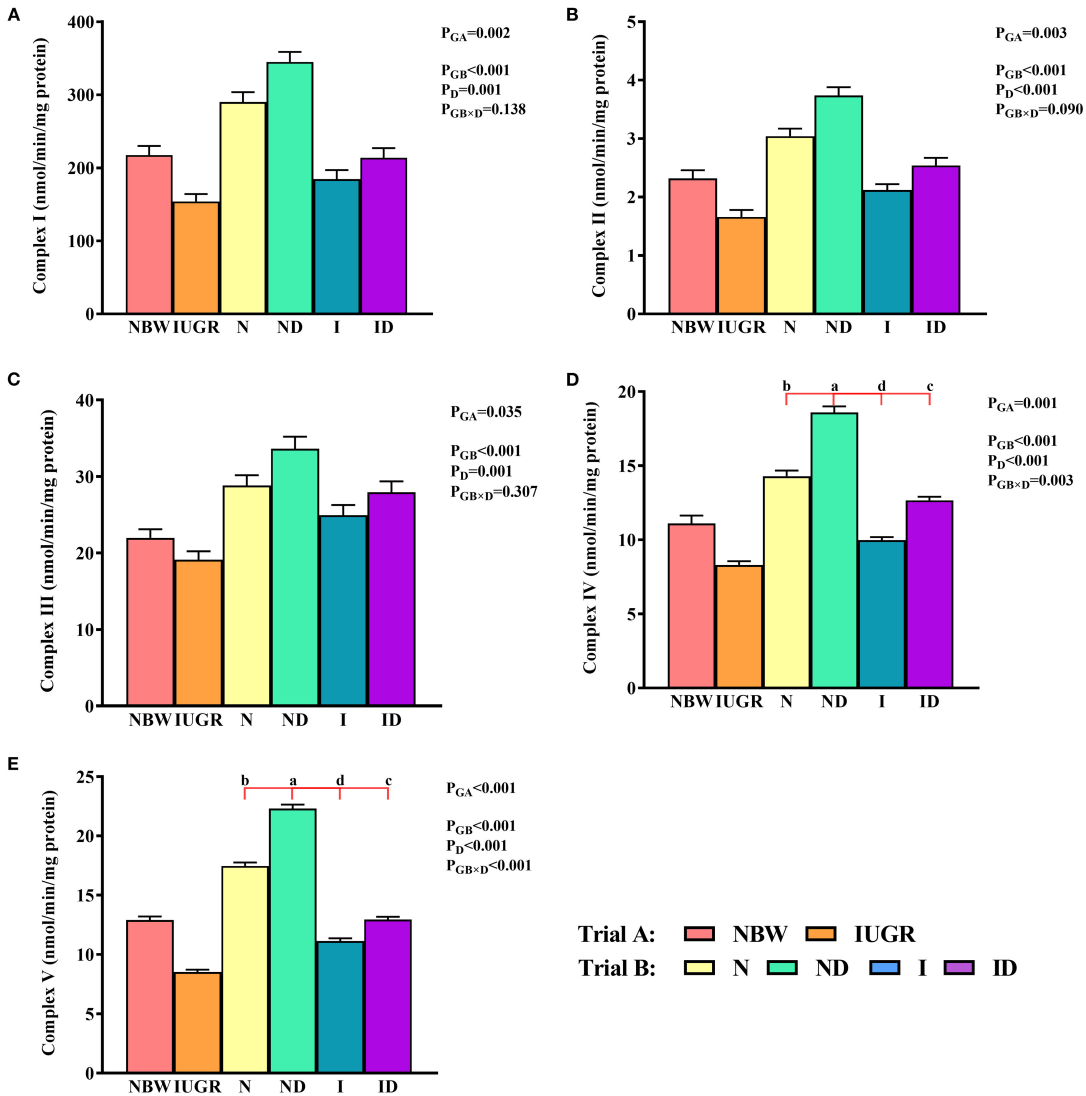
**(A–E)** Supplementation with DMG-Na improved the hepatic mitochondrial ETC complexes in IUGR suckling piglets. Data are expressed as the mean ± SD; *n* = 10 suckling piglets per group. The *P*-value (Trial A: NBW, IUGR group in black color) and a, b, c, d (Trial B: N, ND, I, ID group in red color) represent significant differences (*P* < 0.05). NBW, normal birth weight newborns; IUGR, intrauterine growth restriction newborns; N, NBW newborns fed a basic milk diet; ND, NBW newborns fed a basic milk diet plus 0.1% DMG-Na; I, IUGR newborns fed a basic milk diet; ID, IUGR newborns fed a basic milk diet plus 0.1% DMG-Na.

In trial A (Figure 6), the mitochondrial ETC complexes I–V values in the IUGR group were lower (*P* < 0.05) than were those in the NBW group. In trial B (Figure 6), the interaction effects (groups × DMG-Na) were significant for mitochondrial ETC complex IV (*P* = 0.003) and V (*P* < 0.001). Hepatic mitochondrial ETC complex IV and V values were increased (*P* < 0.05) in the ND and ID groups relative to those in the *N* and I groups, respectively. Additionally, hepatic mitochondrial ETC complex I, IV, and V values were decreased (*P* < 0.05) in the I group relative to those in the *N* group.

### Mitochondrial Energy Metabolism Study

**Figure 7:**
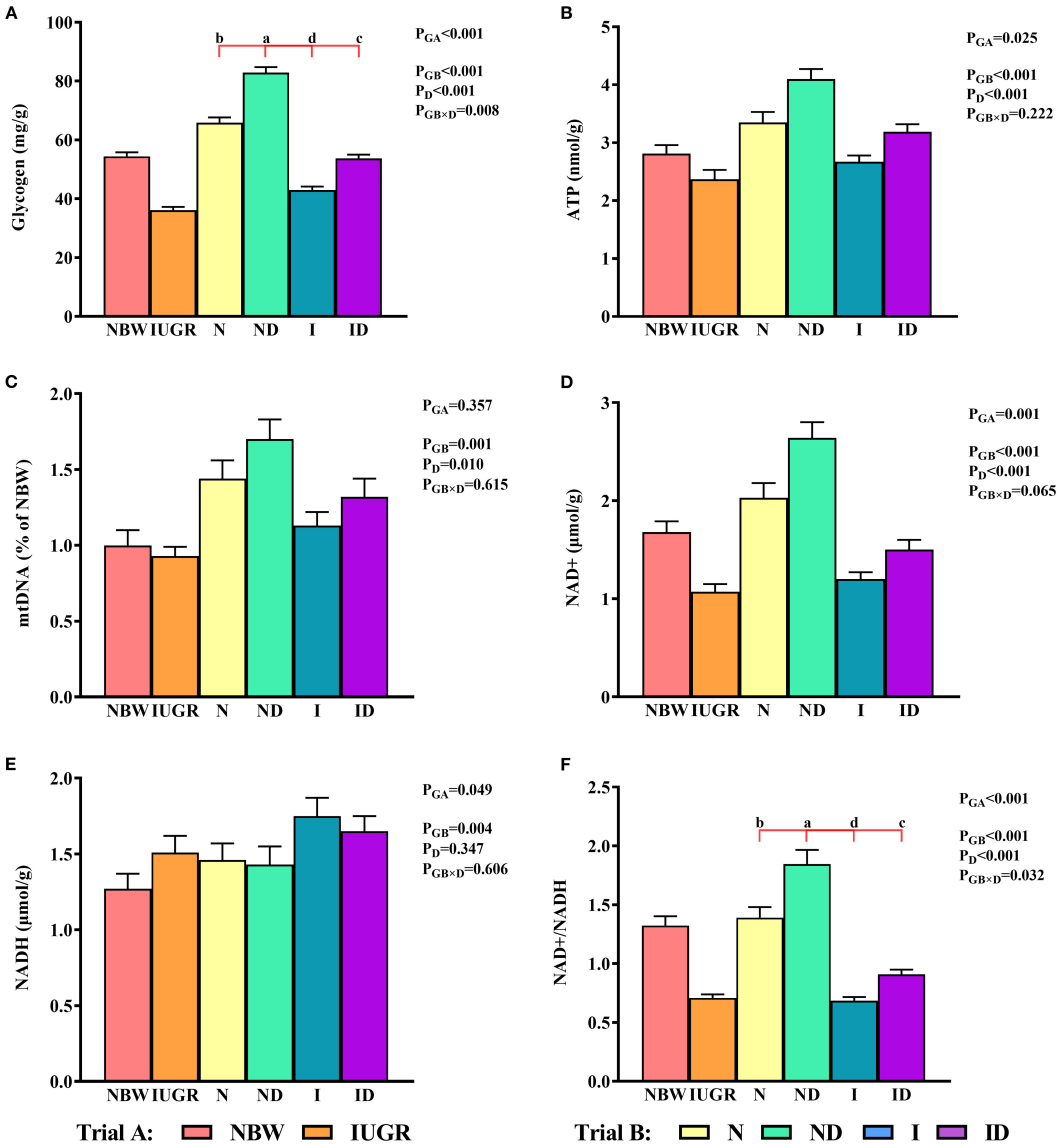
**(A–F)** Supplementation with DMG-Na improved the hepatic energy metabolism in IUGR suckling piglets. Data are expressed as the mean ± SD; *n* = 10 suckling piglets per group. The *P*-value (Trial A: NBW, IUGR group in black color) and a, b, c, d (Trial B: N, ND, I, ID group in red color) represent significant differences (*P* < 0.05). NBW, normal birth weight newborns; IUGR, intrauterine growth restriction newborns; N, NBW newborns fed a basic milk diet; ND, NBW newborns fed a basic milk diet plus 0.1% DMG-Na; I, IUGR newborns fed a basic milk diet; ID, IUGR newborns fed a basic milk diet plus 0.1% DMG-Na; ATP, adenosine triphosphate; NAD^+^, nicotinamide adenine dinucleotide; NADH, nicotinamide-adenine dinucleotide.

In trial A (Figure 7), the mitochondrial glycogen, ATP, NAD^+^, NADH, and NAD^+^/NADH values in the IUGR group were lower (*P* < 0.05) than those in the NBW group. In trial B (Figure 7), the interaction effects (groups × DMG-Na) were significant for glycogen (*P* = 0.008) and NAD^+^/NADH (*P* = 0.032). Hepatic glycogen and NAD^+^/NADH values were increased (*P* < 0.05) in the ND and ID groups relative to those in the N and I groups, respectively. Additionally, hepatic glycogen values were lower (*P* < 0.05) in the I group than they were in the N group.

### Gene Expression Study

**Figure 8:**
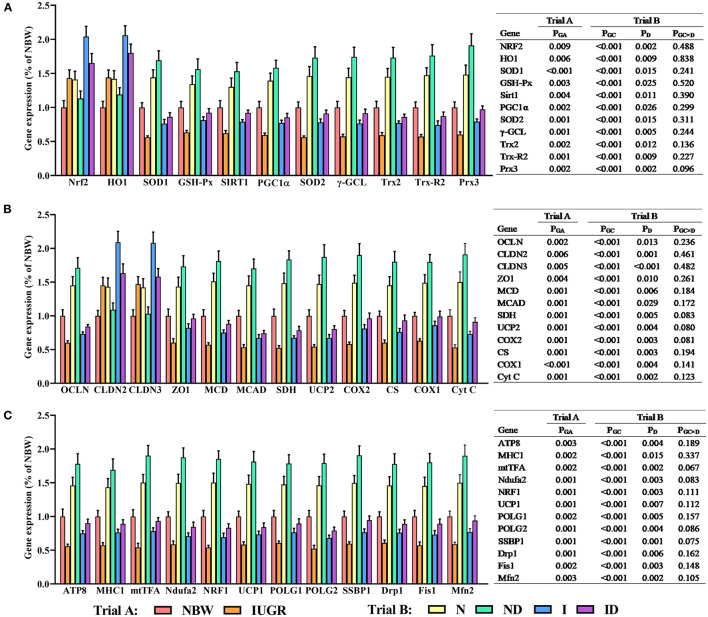
**(A–C)** Supplementation with DMG-Na improved the hepatic gene expression values in IUGR suckling piglets. Data are expressed as the mean ± SD; *n* = 10 suckling piglets per group. The *P*-value (Trial A: NBW, IUGR group in black color) and a, b, c, d (Trial B: N, ND, I, ID group in red color) represent significant differences (*P* < 0.05). NBW, normal birth weight newborns; IUGR, intrauterine growth restriction newborns; N, NBW newborns fed a basic milk diet; ND, NBW newborns fed a basic milk diet plus 0.1% DMG-Na; I, IUGR newborns fed a basic milk diet; ID, IUGR newborns fed a basic milk diet plus 0.1% DMG-Na. Nrf2, nuclear factor erythroid 2-related factor 2; HO1, heme oxygenase 1; SOD1, copper and zinc superoxide dismutase; GSH-Px, glutathione peroxidase; Sirt1, sirtuin 1; PGC1α, peroxisome proliferator-activated receptorγcoactivator-1α; SOD2, manganese superoxide dismutase; γ-GCL, γ-glutamylcysteine ligase; Trx2, thioredoxin 2; Trx-R2, thioredoxin reductase 2; Prx3, peroxiredoxin 3; OCLN, occluding; CLDN2, cloudin2; CLDN3, cloudin3; ZO1, zonula occludens-1; MCD, lipid oxidation enzymes malonyl-CoA decarboxylase; MCAD, medium-chain acyl-CoA dehydrogenase; SDH, mitochondrial proteins succinate dehydrogenase; UCP2, uncoupling protein 2; COX2, cyclooxygenase 2; CS, citrate synthase; COX1, cyclooxygenase 1; Cyt C, Cytochrome C; MHC1, major histocompatibility complex I; mtTFA, mitochondrial transcription factor A; Ndufa2, NADH dehydrogenase (ubiquinone) iron-sulfur protein 2; NRF1, nuclear respiratory factor 1; UCP1, uncoupling protein 1; POLG1, γ DNA polymerases catalytic subunit γ; POLG2, DNA polymerases accessory subunit; SSBP1, single-strand DNA binding protein 1; Drp1, dynamin-related protein 1; Fis1, mitochondrial fission 1; Mfn2, mitochondrial mitofusin2.

In trial A (Figure 8), all hepatic gene expressions in IUGR newborns were worse (*P* < 0.05) compared to those in NBW newborns. In trial B (Figure 8), the interaction effects (groups × DMG-Na) were not significant (*P* > 0.05) for any of the tested gene expression traits. Additionally, the expression of selected genes in the liver was worse (*P* < 0.05) in the I group compared to that in the N group.

### Western Blot Study

**Figure 9:**
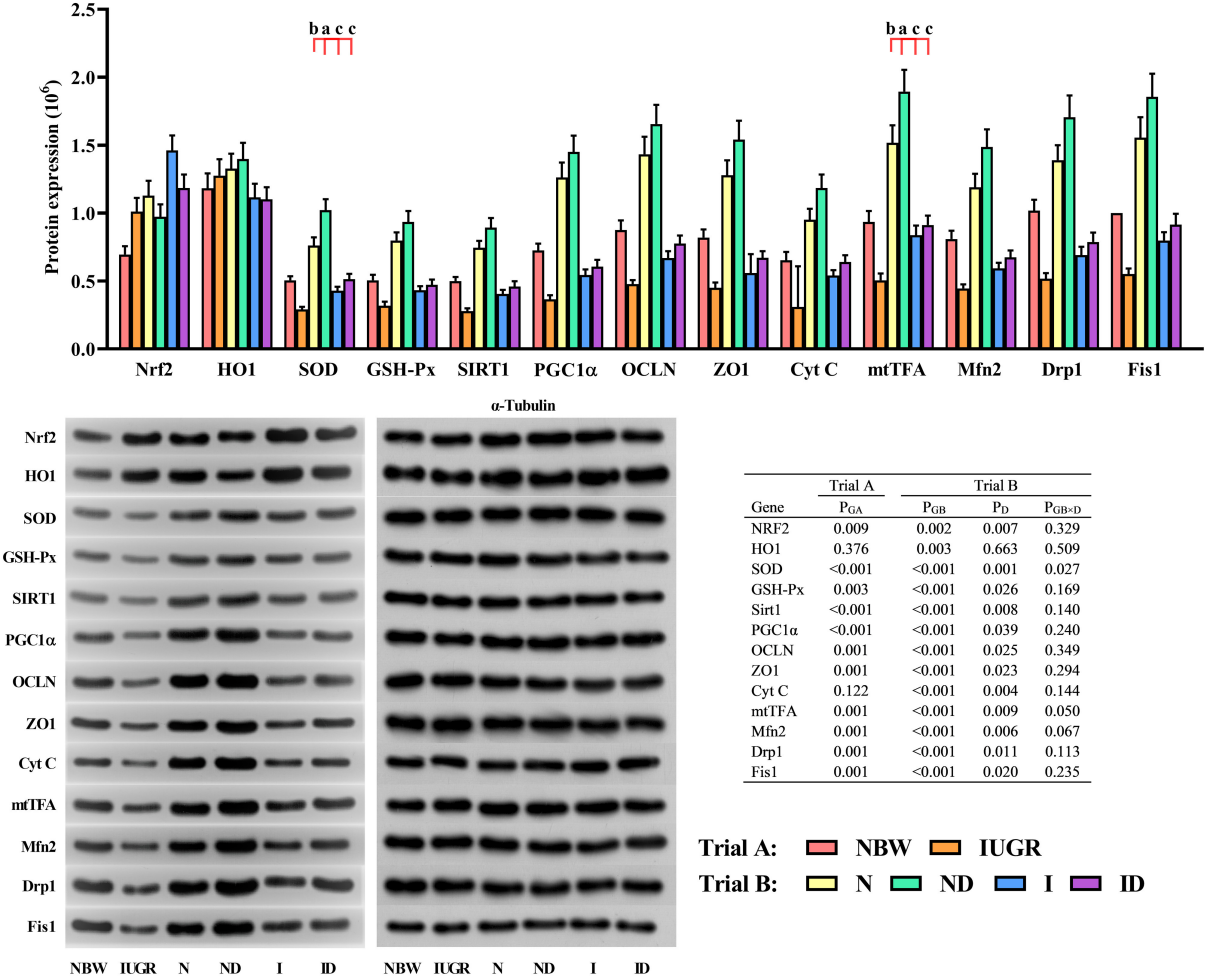
Supplementation with DMG-Na improved the hepatic protein expression values in IUGR suckling piglets. Data are expressed as the mean ± SD, *n* = 10 suckling piglets per group. The *P*-value (Trial A: NBW, IUGR group in black color) and a, b, c, d (Trial B: N, ND, I, ID group in red color) represent significant differences (*P* < 0.05). NBW, normal birth weight newborns; IUGR, intrauterine growth restriction newborns; N, NBW newborns fed a basic milk diet; ND, NBW newborns fed a basic milk diet plus 0.1% DMG-Na; I, IUGR newborns fed a basic milk diet; ID, IUGR newborns fed a basic milk diet plus 0.1% DMG-Na. Nrf2, nuclear factor erythroid 2-related factor 2; HO1, heme oxygenase 1; SOD, superoxide dismutase; GSH-Px, glutathione peroxidase; Sirt1, sirtuin 1; PGC1α, peroxisome proliferator-activated receptorγcoactivator-1α; OCLN, occluding; ZO1, zonula occludens-1; Cyt C, Cytochrome C; mtTFA, mitochondrial transcription factor A; Mfn2, mitochondrial mitofusin2; Drp1, dynamin-related protein 1; Fis1, mitochondrial fission 1.

In trial A (Figure 9), the gene expression in the liver of IUGR newborns was worse (*P* < 0.05) compared to that in NBW newborns. In trial B (Figure 9), the interaction effects (groups × DMG-Na) were significant for SOD (*P* = 0.027) and mtTFA (*P* = 0.050). Protein expression in the liver was better (*P* < 0.05) in the ND and ID groups relative to that in the *N* and I groups, respectively. Additionally, the selected protein expression in the liver was worse (*P* < 0.05) in the I group than was in the *N* group.

## Discussion

Intrauterine growth restriction is one most important problem in animal husbandry, as it can impair postnatal growth and liver function by irreversible oxidative damage (18). Several studies have been performed on IUGR piglets as a model to present poor performance (1, 2, 18), and they are in agreement with the present study using newborns with birth weights of 1.53 ± 0.04 kg and 0.76 ± 0.06 kg as NBW newborns and IUGR newborns, respectively. The liver is one of the most important organs that play an essential role in the transformation of nutrients. It is also responsible for metabolic homeostasis, therefore, impaired by oxidative damage easily (19). In our previous studies, DMG-Na was found to exert strong free radical scavenging capacity *in vitro* and prove to ameliorate the oxidative damage induced by LPS or indomethacin in mice (10). In addition, DMG-Na was beneficial in protecting AAPH-induced porcine erythrocyte hemolysis, preventing oleic acid hydroperoxides-induced IPEC-J2 damage, and improving the skeletal muscle stem cells' mitochondrial dysfunction by Nrf2/SIRT1/PGC1α network (11, 20). It also revealed that dietary supplementation of DMG-Na could improve the low growth performance, skeletal structure damage, and skeletal muscle dysfunction of IUGR weaned piglets (12). In this study, the average daily body weight gain of I group (0.44) was higher than N group (0.35) at 19–21 d, which might be explained by the 1.5 times higher feed intake of IUGR suckling piglets than that of the NBW suckling piglets during 19–21 d. The metabolic function of the liver under the dietary supplementation of DMG Na could provide conditions for catch-up growth of IUGR offspring (21, 22). However, from the body weight data (Supplementary Table S2), we can see that although feed intake and daily body weight gain of IUGR piglets have increased, their body weight is still at a low level, which still needs more work on this in the future. In addition, supplementation with DMG-Na could improve the growth performance of IUGR suckling piglets compared to those of NBW suckling piglets. This is probably due to the benefits of DMG-Na on liver health by improving their antioxidant capacity and confirmed by the ALT and AST concentration. Taken together, DMG-Na could act as a health-promoting additive for piglets, however, the specific underlying mechanism requires further study.

Oxidative damage of suckling piglets caused by IUGR could enhance the ROS concentration, thus, in turn, destroying the balance of redox status and impairing mitochondrial function. This could be improved in this study by SOD that catalyzes the conversion of endogenous superoxide anions to hydrogen peroxide through disproportionation, and hydrogen peroxide is then finally neutralized by intracellular CAT and GSH-Px. The MnSOD, GSH, GR, and γ-GCL are important in inhibiting mitochondrial oxidative damage (23). Several studies revealed that DMG-Na, shown as an antioxidant additive, could improve the redox status of the animal, thus improving the ROS-induced oxidative damage (10–12, 24). In the current study, DMG-Na added to the basic milk diets could be beneficial in improving the redox status by scavenging the excessive generated ROS, thus maintaining the hepatic mitochondrial function. This would be one main possible reason for the results shown in the histological analysis.

The ROS content in the body maintains a balance with the antioxidant defense system, this could be destroyed when the body is under a condition of oxidative stress. Excessive generated ROS can damage the structure and function of mitochondria, and finally, disturb the balance of redox status (25). Intrauterine growth restriction is closely related to oxidative damage, high ROS concentration, reduced redox status, and mitochondrial dysfunction (24). It was found that excessive generated ROS could induce mtDNA damage, which impaired mitochondrial function in turn produces more ROS (26). The MMP level is negatively associated with ROS concentration and usually acts as an indicator of mitochondrial apoptosis. In agreement with the present study, another study revealed that IUGR destroyed the redox status balance primarily due to the high ROS generation (27). A previous study also showed that IUGR newborns exhibit redox status imbalance and are easily prone to oxidative damage (1). The current results revealed that the reduced redox status in suckling piglets from the IUGR group leads to impaired hepatic mitochondrial function. These results also indicated that DMG-Na added in the basic milk diets could relieve oxidative damage by reducing the excessive generated ROS in suckling piglets, and numeral studies have found that natural antioxidants exert beneficial effects in protecting cells from oxidative damage (10–12, 24). The present results could be explained that supplementation with DMG-Na reduced hepatic oxidative damage by directly reducing the ROS generation and indirectly strengthening the mitochondrial function.

Adenosine triphosphate (ATP) concentration can be measured quantitatively using the mtDNA copy number and are necessary for growth and glycogen synthesis and are related to the mitochondrial number. A lower ATP content in IUGR suckling piglets leads to lower postnatal hepatic glycogen storage (12). The loss activity of complex I in the hepatic mitochondria of IUGR suckling piglets can decrease mitochondrial energy generation. In the present study, the lower NAD^+^/NADH ratio could be explained by the reduced complex I activity, which couples electrons from NADH to quinone with the translocation of a proton across the inner mitochondrial membrane for ATP generation (28). In addition, it suppresses the flux of glycolytic and tricarboxylic acid cycle metabolites by the mitochondria, therefore leading to reduced generation of ATP (28). An increase in complex IV or V activity in the hepatic mitochondria of IUGR suckling piglets is important in bioenergetics, a process that uses the exergonic proton backflow for ATP synthesis from ADP and inorganic phosphate within the matrix (29). It has been found that DMG-Na as an antioxidant additive could protect the liver from oxidative damage and maintain its function. A previous study revealed that DMG-Na exerted a beneficial effect on protecting the cells from oxidative damage induced by free radicals (24), and this might be one main reason for improving the hepatic mitochondrial ETC complex activity and energy metabolism level in the DMG-Na supplemented group than the DMG-Na non-supplemented group.

The Nrf2 and HO1 activation is crucial in inhibiting hepatic oxidative damage by regulating redox status-related gene expression (*SOD, GSH-Px*, and γ*-GCL*) (30). Hepatic mitochondria are rich in *Trx2, Trx-R2*, and *Prx3* that act in concert to prevent oxidative damage by scavenging excessive generated free radicals and regulating mitochondrial redox status-related gene expression (31). PGC1α is a coactivator possessing pleiotropic functions that regulate mitochondrial function gene expression (*MCD, MCAD, SDH, UCP2, COX2, CS, COX1, Cyt C, mtTFA, Ndufa2, UCP1, mtDNA, POLG1, POLG2, Drp1, Fis1*, and *Mfn2*) both at the level of the nuclear and mitochondrial genomes. SIRT1 is highly sensitive to cellular redox status and is famous for controlling genomic stability (32). It has been found that SIRT1 physically interacts with and deacetylates PGC1α at multiple lysine sites, consequently increasing PGC1α activity and regulating the redox status-related and mitochondrial function-related gene and protein expression (33). The ZO1, correlated with paracellular permeability and acts together with OCLN and CLDN, is an important regulator of cell permeability (34). To our knowledge, this is the first study on supplementation with dimethylglycine sodium salt relieves hepatic redox status imbalance and mitochondrial dysfunction of intrauterine growth restriction suckling piglets *via* the Nrf2/SIRT1/PGC1α network. However, there still needs more work on the specific underlying mechanism.

## Conclusions

In conclusion, this study revealed that DMG-Na could reverse hepatic dysfunction in IUGR suckling piglets. We also found that DMG-Na could directly neutralize excessive generated free radicals and indirectly improve the redox status-related and mitochondrial function-related gene and protein expression possibly *via* the Nrf2/SIRT1/PGC1α network. Thus, DMG-Na can be acted as a health-promoting additive in treating hepatic dysfunction in IUGR suckling piglets and is beneficial in improving their performance during the suckling period.

## Data Availability Statement

The original contributions presented in the study are included in the article/Supplementary Material, further inquiries can be directed to the corresponding author.

## Ethics Statement

This trial was conducted in accordance with the Chinese Guidelines for Animal Welfare and Experimental Protocols for Animal Care and was approved by the Nanjing Agricultural University Institutional Animal Care and Use Committee.

## Author Contributions

KB and LJ performed the experiments. KB analyzed the data and wrote the original manuscript. TW obtained the funding, contributed to the study design, and revised the manuscript. All authors reviewed the final manuscript.

## Funding

This work was supported by the National Natural Science Foundation of China (31572418 and 31802101) and National Key Research and Development Program of China (2018YFD0501101).

## Conflict of Interest

The authors declare that the research was conducted in the absence of any commercial or financial relationships that could be construed as a potential conflict of interest.

## Publisher's Note

All claims expressed in this article are solely those of the authors and do not necessarily represent those of their affiliated organizations, or those of the publisher, the editors and the reviewers. Any product that may be evaluated in this article, or claim that may be made by its manufacturer, is not guaranteed or endorsed by the publisher.

## References

[1] Sacchi C Marino C Nosarti C Vieno A Visentin S Simonelli A. Association of intrauterine growth restriction and small for gestational age status with childhood cognitive outcomes: a systematic review and meta-analysis. JAMA Pediatr. (2020) 174:772–81. 10.1001/jamapediatrics.2020.1097 32453414 PMC7251506

[2] Guerby P Bujold E. Early detection and prevention of intrauterine growth restriction and its consequences. JAMA Pediatr. (2020) 174:749–50. 10.1001/jamapediatrics.2020.1106 32453430

[3] Alferink LJM Kiefte-de Jong JC Darwish Murad S. Animal protein intake and hepatic steatosis in the elderly: authors' response. Gut. (2020) 69:189. 10.1136/gutjnl-2018-317843 30464046

[4] Pendleton AL Wesolowski SR Regnault TRH Lynch RM Limesand SW. Dimming the powerhouse: mitochondrial dysfunction in the liver and skeletal muscle of intrauterine growth restricted fetuses. Front Endocrinol. (2021) 12:612888. 10.3389/fendo.2021.612888 34079518 PMC8165279

[5] Bock FJ Tait SWG. Mitochondria as multifaceted regulators of cell death. Nat Rev Mol Cell Biol. (2020) 21:85–100. 10.1038/s41580-019-0173-8 31636403

[6] He F Antonucci L Yamachika S Zhang Z Taniguchi K Umemura A. Nrf2 activates growth factor genes and downstream akt signaling to induce mouse and human hepatomegaly. J Hepatol. (2020) 72:1182–95. 10.1016/j.jhep.2020.01.023 32105670 PMC8054878

[7] Liu J Du S Kong Q Zhang X Jiang S Cao X. Hspa12a attenuates lipopolysaccharide-induced liver injury through inhibiting caspase-11-mediated hepatocyte pyroptosis via pgc-1α-dependent acyloxyacyl hydrolase expression. Cell Death Differ. (2020) 27:2651–67. 10.1038/s41418-020-0536-x 32332915 PMC7429872

[8] Lee SE Koh H Joo DJ Nedumaran B Jeon HJ Park CS. Induction of sirt1 by melatonin improves alcohol-mediated oxidative liver injury by disrupting the crbn-yy1-cyp2e1 signaling pathway. J Pineal Res. (2020) 68:e12638. 10.1111/jpi.12638 32053237

[9] Rodgers JT Lerin C Haas W Gygi SP Spiegelman BM Puigserver P. Nutrient control of glucose homeostasis through a complex of pgc-1alpha and sirt1. Nature. (2005) 434:113–8. 10.1038/nature03354 15744310

[10] Bai K Jiang L Zhu S Feng C Zhao Y Zhang L. Dimethylglycine sodium salt protects against oxidative damage and mitochondrial dysfunction in the small intestines of mice. Int J Mol Med. (2019) 43:2199–211. 10.3892/ijmm.2019.4093 30816456

[11] Bai K Jiang L Zhang L Zhao Y Lu Y Zhu J. In vitro free radical scavenging capacity of dimethylglycine sodium salt and its protective ability against oleic acid hydroperoxide-induced oxidative damage in ipec-j2 cells. Int J Mol Med. (2018) 42:3447–58. 10.3892/ijmm.2018.3876 30221672

[12] Bai K Jiang L Li Q Zhang J Zhang L Wang T. Dietary dimethylglycine sodium salt supplementation improves growth performance, redox status, and skeletal muscle function of intrauterine growth-restricted weaned piglets. J Anim Sci. (2021) 99:skab186. 10.1093/jas/skab186 34107017 PMC8290491

[13] Wang T Huo YJ Shi F Xu RJ Hutz RJ. Effects of intrauterine growth retardation on development of the gastrointestinal tract in neonatal pigs. Biol Neonate. (2005) 88:66–72. 10.1159/000084645 15785017

[14] Dong L Zhong X He J Zhang L Bai K Xu W. Supplementation of tributyrin improves the growth and intestinal digestive and barrier functions in intrauterine growth-restricted piglets. Clin Nutr. (2016) 35:399–407. 10.1016/j.clnu.2015.03.002 26112894

[15] Liu L Xie B Fan M Candas-Green D Jiang JX Wei R. Low-level saturated fatty acid palmitate benefits liver cells by boosting mitochondrial metabolism via cdk1-sirt3-cpt2 cascade. Dev Cell. (2020) 52:196–209. 10.1016/j.devcel.2019.11.012 31866205 PMC6996588

[16] Zhang Q Zou P Zhan H Zhang M Zhang L Ge RS. Dihydrolipoamide dehydrogenase and camp are associated with cadmium-mediated leydig cell damage. Toxicol Lett. (2011) 205:183–9. 10.1016/j.toxlet.2011.06.003 21699967

[17] Livak KJ Schmittgen TD. Analysis of relative gene expression data using real-time quantitative PCR and the 2[-delta delta c(t)] method. Methods. (2001) 25:402–8. 10.1006/meth.2001.1262 11846609

[18] Sovio U Goulding N McBride N Cook E Gaccioli F Charnock-Jones DS. A maternal serum metabolite ratio predicts fetal growth restriction at term. Nat Med. (2020) 26:348–53. 10.1038/s41591-020-0804-9 32161413

[19] Michalopoulos GK Bhushan B. Liver regeneration: biological and pathological mechanisms and implications. Nat Rev Gastroenterol Hepatol. (2021) 18:40–55. 10.1038/s41575-020-0342-4 32764740

[20] Bai K Jiang L Wei C Li Q Zhang L Zhang J. Dimethylglycine sodium salt activates Nrf2/SIRT1/PGC1α leading to the recovery of muscle stem cell dysfunction in newborns with intrauterine growth restriction. Free Radic Biol Med. (2022) 184:89–98. 10.1016/j.freeradbiomed.2022.04.004 35405266

[21] Darendeliler F IUGR. Genetic influences, metabolic problems, environmental associations/triggers, current and future management. Best Pract Res Clin Endocrinol Metab. (2019) 33:101260. 10.1016/j.beem.2019.01.001 30709755

[22] Heber MF Ptak GE. The effects of assisted reproduction technologies on metabolic health and disease. Biol Reprod. (2021) 104:734–44. 10.1093/biolre/ioaa224 33330924 PMC8023432

[23] Li S Li H Xu X Saw PE Zhang L. Nanocarrier-Mediated antioxidant delivery for liver diseases. Theranostics. (2020) 10:1262–80. 10.7150/thno.38834 31938064 PMC6956819

[24] Feng C Bai K Wang A Ge X Zhao Y Zhang L. Effects of dimethylglycine sodium salt supplementation on growth performance, hepatic antioxidant capacity, and mitochondria-related gene expression in weanling piglets born with low birth weight1. J Anim Sci. (2018) 96:3791–803. 10.1093/jas/sky233 29931075 PMC6127790

[25] Zhou H Du W Li Y Shi C Hu N Ma S. Effects of melatonin on fatty liver disease: the role of nr4a1/dna-pkcs/p53 pathway, mitochondrial fission, and mitophagy. J Pineal Res. (2018) 64. 10.1111/jpi.12450 28981157

[26] Ni D Wei H Chen W Bao Q Rosenkrans ZT Barnhart TE. Ceria nanoparticles meet hepatic ischemia-reperfusion injury: the perfect imperfection. Adv Mater. (2019) 31:e1902956. 10.1002/adma.201902956 31418951 PMC6773480

[27] Zhu HL Shi XT Xu XF Zhou GX Xiong YW Yi SJ. Melatonin protects against environmental stress-induced fetal growth restriction via suppressing ros-mediated gcn2/atf4/bnip3-dependent mitophagy in placental trophoblasts. Redox Biol. (2021) 40:101854. 10.1016/j.redox.2021.101854 33454563 PMC7811044

[28] Kampjut D Sazanov LA. The coupling mechanism of mammalian respiratory complex I. Science. (2020) 370:eabc4209. 10.1126/science.abc4209 32972993

[29] Giacomello M Pyakurel A Glytsou C Scorrano L. The cell biology of mitochondrial membrane dynamics. Nat Rev Mol Cell Biol. (2020) 21:204–24. 10.1038/s41580-020-0210-7 32071438

[30] Saeedi BJ Liu KH Owens JA Hunter-Chang S Camacho MC Eboka RU. Gut-resident lactobacilli activate hepatic nrf2 and protect against oxidative liver injury. Cell Metab. (2020) 31:956–68. 10.1016/j.cmet.2020.03.006 32213347 PMC7329068

[31] Upadhyay KK Jadeja RN Vyas HS Pandya B Joshi A Vohra A. Carbon monoxide releasing molecule-a1 improves nonalcoholic steatohepatitis via nrf2 activation mediated improvement in oxidative stress and mitochondrial function. Redox Biol. (2020) 28:101314. 10.1016/j.redox.2019.101314 31514051 PMC6737302

[32] Collin de l'Hortet A Takeishi K Guzman-Lepe J Morita K Achreja A Popovic B. Generation of human fatty livers using custom-engineered induced pluripotent stem cells with modifiable sirt1 metabolism. Cell Metab. (2019) 30:385–401. 10.1016/j.cmet.2019.06.017 31390551 PMC6691905

[33] Wang S Wan T Ye M Qiu Y Pei L Jiang R. Nicotinamide riboside attenuates alcohol induced liver injuries via activation of sirt1/pgc-1α/mitochondrial biosynthesis pathway. Redox Biol. (2018) 17:89–98. 10.1016/j.redox.2018.04.006 29679894 PMC6007172

[34] Amarachintha SP Mourya R Ayabe H Yang L Luo Z Li X. Biliary organoids uncover delayed epithelial development and barrier function in biliary atresia. Hepatology. (2021). 75:89–103. 10.1002/hep.32107 34392560 PMC9983428

