# Supplementary material for: Dimethylglycine Sodium Salt Alleviates Intrauterine Growth Restriction-Induced Low Growth Performance, Redox Status Imbalance, and Hepatic Mitochondrial Dysfunction in Suckling Piglets
Source: Front Vet Sci. 2022 Jun 24;9:905488. doi: 10.3389/fvets.2022.905488 (PMC9263627; doi:10.3389/fvets.2022.905488)
Supplement: Supplementary file 1 [file Table_1.docx]

Supplementary Material

**Table S1.** Primer sequences used for Real-time PCR assay

| Gene^1^ | Primer sequence^2^ | Genbank ID^3^ | Gene^1^ | Primer sequence^2^ | Genbank ID^3^ |
| --- | --- | --- | --- | --- | --- |
| *β-actin* | GTTCGAGACCTTCAACACGC | XM_003357928.3 | *SDH* | ATGGAAAACGGGGAGTGTCG | CX063991.1 |
|  | CCATGACAATGCCAGTGGTG |  |  | TTCCGGTAGCGACAACAGTG |  |
| *Nrf2* | GACAAACCGCCTCAACTCAG | NM_001114671.1 | *UCP2* | CGTGGTAAAGGTCCGGTTC | NM_214289.1 |
|  | GTCTCCACGTCGTAGCGTTC |  |  | AGGAGCGTGTCCTTGATGAG |  |
| *HO1* | ACACTGGGCTGGAGAGATCA | NM_001138825.2 | *COX2* | GAGACAGCATAAACTGCGCC | NM_214321.1 |
|  | AGAGTAGCGTATGTGGTGCC |  |  | AGTGTCTTTGGCTGTCGGAG |  |
| *SOD1* | AGGGAGAGAAGACAGTGTTAGT | NM_001190422.1 | *CS* | CCACAGTGACCATGAAGGTG | NM_214276.1 |
|  | GTACACAGTGGCCACACCAT |  |  | AACCCGTCCTGAGTTGAGTG |  |
| *GSH-Px* | CTTATGGTGGGGTAGGGGGA | XM_003463071.2 | *COX1* | GCAGTTGCCAGATGCTGAAC | XM_001926129.5 |
|  | GGCTGGGTAGGAGATTGCAG |  |  | TGGGTGAAGTGTTGGGCAAA |  |
| *SOD2* | GGCGCCATCAAGTTCAACAG | NM_001281206.1 | *Cyt C* | TCGGAGTCACCAGTGCTAGA | XM_003127002.3 |
|  | TGAGCCTTTTGGGGGTTCAC |  |  | GGAACATTGAGGCCTACGGA |  |
| *γ-GCLc* | CCCTTGTGGTACCTCTGCAT | XM_013977739.1 | *ERRα* | GGTGGGCGACAGAAGTACAA | NM_001170521.1 |
|  | TGGGTTTGTCCTTTCCCCCT |  |  | ACCACAATCTCTCGGTCGAA |  |
| *γ-GCLm* | AAGGGAAGTGGAGTCATGCG | XM_001926378.3 | *MHCI* | GAAGCCTTCTGGACACCTTCA | AF013960.1 |
|  | AAGGCTAGCTTCTTCGAGGG |  |  | CCAGAACCAGGCCAACAGT |  |
| *Trx2* | GGACCGAATCTGAGCCAAGT | NM_123358.4 | *mtTFA* | TATAGGGCAGACTGGCAGGT | NM_001130211.1 |
|  | GCACCATGAGGCCGAGAAAT |  |  | TGGACCATCCTTAGCTTCCT |  |
| *Trx-R2* | GATACCCCACGCACATCGAA | NM_001282512.1 | *Ndufa2* | TGCTAAGTGGCAAAGCCTGA | XM_003124046.3 |
|  | CCCGACCACCAACGTTTTTC |  |  | GAACAAAACATCCGGAGGCG |  |
| *Prx3* | CAATGTCGACCGCAAGAACG | NM_079663.3 | *NRF1* | TTGCAGAGGTGCAATCAAATGG | XM_013985624.1 |
|  | GTCAATGATGAAGGTGCCGC |  |  | TCCAAAATCCGCTGCCTTTC |  |
| *Sirt1* | GGTGGTTCCTCGATGTCCTA | NM_001145750.1 | *UCP1* | AAGCAAGGAGCATTCCTGGG | XM_013978885.1 |
|  | GGTGAGGCAAAGGTTCCCTA |  |  | CTGCCCTGTGGGTAACCATT |  |
| *PGC1α* | GGGGCCCATGGGAATCATC | XM_013992150.1 | *POLG1* | AGCAGAAGCCCCAAAGTTCC | XM_001927064.4 |
|  | AACTGCTGTTGTTTGGGCCT |  |  | AGCATGACCTCTCTCCCTTCT |  |
| *OCLN* | CCTCCTCCCCTTTCGGACTA | NM_001163647 | *POLG2* | AGCCCCAAAGTTCCCATTAACT | XM_013997017.1 |
|  | TAGACCCCTAGCCTGGGAAC |  |  | ACCTCTCTCCCTTCTAGGGT |  |
| *CLDN2* | ATCAAGCAAGGGCAGAAACG | XM_021079578 | *SSBP1* | CATACCAAATGGGCGATGTC | XM_013985577.1 |
|  | GGATGCGATTGGTGGGTTTG |  |  | TTGTGGTTGCTTGTCGTCTC |  |
| *CLDN3* | TGTCCGTCTATCCGTCCGTC | NM_001160075 | *Mfn2* | TAGTAGGGTCGAACACGCTG | XM_021095371 |
|  | ATCCGCGCTGTGATAATGCT |  |  | GATGCCCCTCACTTTGGACA |  |
| *ZO1* | CTGCCAAGTGAAACTGCACA | AJ318101 | *Drp1* | AAGAGGAGTCGCATGTGTCG | XM_001928848 |
|  | GACAGAGAACGTGTCAACGC |  |  | TGCAATCATGCCCACCAGAT |  |
| *MCD* | ACAGATGTGAGGCTCTGTGC | FJ263687 | *Fis1* | AGTAGTGAGGATTGCGAGGC | XM_021086263 |
|  | CTCATGTCTGGATCTCCGGC |  |  | TACTTGCTTCGCACCAGACA |  |
| *MCAD* | AAACCAGACCTTCGGTAGCA | NM_214039.1 |  |  |  |
|  | GTATTTCGGCGACCAGAATC |  |  |  |  |

^1^ Nrf2, nuclear factor erythroid 2-related factor 2; HO1, heme oxygenase 1; Cu/ZnSOD, copper and zinc superoxide dismutase; GSH-Px, glutathione peroxidase; MnSOD, manganese superoxide dismutase; γ-GCLc, γ-glutamylcysteine ligase c; γ-GCLm, γ-glutamylcysteine ligase m; Trx2, thioredoxin 2; Trx-R2, thioredoxin reductase 2; Prx3, peroxiredoxin 3; Sirt1, sirtuin 1; PGC1α, peroxisome proliferator-activated receptorγcoactivator-1α; OCLN, occluding; CLDN2, cloudin2; CLDN3, cloudin3; ZO1, zonula occludens-1; MCD, lipid oxidation enzymes malonyl-CoA decarboxylase; MCAD, medium-chain acyl-CoA dehydrogenase; SDH, mitochondrial proteins succinate dehydrogenase; UCP2, uncoupling protein 2; COX2, cyclooxygenase 2; CS, citrate synthase; COX1, cyclooxygenase 1; Cyt C, Cytochrome C; ERRα, estrogen-related receptor a; MHC1, major histocompatibility complex I; mtTFA, mitochondrial transcription factor A; Ndufa2, NADH dehydrogenase (ubiquinone) iron-sulfur protein 2; NRF1, nuclear respiratory factor 1; UCP1, uncoupling protein 1; POLG1, γ DNA polymerases catalytic subunit; POLG2, γ DNA polymerases accessory subunit; SSBP1, single-strand DNA binding protein 1; Drp1, dynamin-related protein 1; Fis1, mitochondrial fission 1; Mfn2, mitochondrial mitofusin2.

^2^ Shown as forward primer followed by reverse primer.

^3^ GenBank Accession Number.

**Table S2.** Supplementation with DMG-Na improved the growth performance of IUGR suckling piglets^1^

|  | Treatment ^2^ | | | | *P* value | | | | | | |
| --- | --- | --- | --- | --- | --- | --- | --- | --- | --- | --- | --- |
| Item | N | ND | I | ID | *P*_G_ | *P*_T_ | *P*_D_ | *P* _G×T_ | *P* _G×D_ | *P* _T×D_ | *P* _G×T×D_ |
| 0 d | 1.53±0.24^a^ | - | 0.76±0.10^b^ | - | <0.001 | <0.001 | <0.001 | 0.004 | 0.021 | 0.002 | <0.001 |
| 7 d | 3.48±0.26^a^ | - | 2.23±0.21^b^ | - |  |  |  |  |  |  |  |
| 10 d | 3.99±0.35^b^ | 5.03±0.31^a^ | 2.88±0.31^c^ | 3.24±0.35^bc^ |  |  |  |  |  |  |  |
| 13 d | 5.02±0.37^b^ | 6.51±0.36^a^ | 3.48±0.36^c^ | 4.03±0.37^c^ |  |  |  |  |  |  |  |
| 16 d | 6.31±0.46^b^ | 7.45±0.36^a^ | 4.35±0.46^c^ | 5.08±0.45^b^ |  |  |  |  |  |  |  |
| 19 d | 7.01±0.48^b^ | 8.12±0.42^a^ | 5.00±0.50^c^ | 6.15±0.43^b^ |  |  |  |  |  |  |  |
| 21 d | 7.71±0.58^b^ | 8.90±0.52^a^ | 5.88±0.60^c^ | 7.07±0.57^b^ |  |  |  |  |  |  |  |

^1^ Values are expressed as Mean ± SD, n = 10. Different superscripts a, b, c (N, ND, I, ID group) represent significant differences (*P* < 0.05).

^2^ NBW, normal birth weight newborns; IUGR, intrauterine growth restriction newborns; N, NBW newborns fed a basic milk diet; ND, NBW newborns fed a basic milk diet plus 0.1% DMG-Na; I, IUGR newborns fed a basic milk diet; ID = IUGR newborns fed a basic milk diet plus 0.1% DMG-Na.
